# Estimating the absolute wealth of households

**DOI:** 10.2471/BLT.14.147082

**Published:** 2015-05-15

**Authors:** Daniel J Hruschka, Drew Gerkey, Craig Hadley

**Affiliations:** a School of Human Evolution and Social Change, Arizona State University, PO Box 872402, Tempe, Arizona, 85287-2402, United States of America (USA).; b Oregon State University, Corvallis, USA.; c Emory University, Atlanta, USA.

## Abstract

**Objective:**

To estimate the absolute wealth of households using data from demographic and health surveys.

**Methods:**

We developed a new metric, the absolute wealth estimate, based on the rank of each surveyed household according to its material assets and the assumed shape of the distribution of wealth among surveyed households. Using data from 156 demographic and health surveys in 66 countries, we calculated absolute wealth estimates for households. We validated the method by comparing the proportion of households defined as poor using our estimates with published World Bank poverty headcounts. We also compared the accuracy of absolute versus relative wealth estimates for the prediction of anthropometric measures.

**Findings:**

The median absolute wealth estimates of 1 403 186 households were 2056 international dollars per capita (interquartile range: 723–6103). The proportion of poor households based on absolute wealth estimates were strongly correlated with World Bank estimates of populations living on less than 2.00 United States dollars per capita per day (*R^2^* = 0.84). Absolute wealth estimates were better predictors of anthropometric measures than relative wealth indexes.

**Conclusion:**

Absolute wealth estimates provide new opportunities for comparative research to assess the effects of economic resources on health and human capital, as well as the long-term health consequences of economic change and inequality.

## Résumé

**Objectif:**

Estimer la richesse absolue des ménages à l'aide de données provenant d'enquêtes démographiques et sanitaires.

**Méthodes:**

Nous avons développé un nouvel indicateur, l'estimation de la richesse absolue, à partir du classement de chaque ménage interrogé en fonction de ses biens matériels et du mode supposé de répartition des richesses entre les différents ménages interrogés. À l'aide de données provenant de 156 enquêtes démographiques et sanitaires menées dans 66 pays, nous avons produit des estimations de la richesse absolue des ménages. Nous avons validé la méthode en comparant la proportion des ménages définis comme pauvres dans nos estimations aux taux de pauvreté publiés par la Banque mondiale. Nous avons également comparé l'exactitude des estimations de la richesse absolue par rapport aux estimations de la richesse relative en vue de la prévision de mesures anthropométriques.

**Résultats:**

Les estimations moyennes de la richesse absolue de 1.403.186 ménages indiquaient 2056 dollars internationaux par personne (intervalle interquartile: 723–6103). Une forte corrélation a été établie entre la proportion des ménages pauvres d'après les estimations de la richesse absolue et l'estimation du nombre de personnes vivant avec moins de 2,00 dollars des États-Unis par personne et par jour (R2 = 0,84) fournie par la Banque mondiale. Les estimations de la richesse absolue se sont révélées de meilleurs indicateurs des mesures anthropométriques que les indices de richesse relative.

**Conclusion:**

Les estimations de la richesse absolue offrent de nouvelles possibilités de recherche comparative pour évaluer les effets des ressources économiques sur le capital santé et humain, ainsi que les conséquences à long terme des changements et inégalités économiques sur la santé.

## Resumen

**Objetivo:**

Estimar la riqueza absoluta de los hogares utilizando los datos de encuestas demográficas y de salud.

**Métodos:**

Se ha elaborado un nuevo sistema métrico, la estimación de la riqueza absoluta, basado en el rango de cada hogar encuestado en función de sus bienes materiales y la supuesta forma de distribución de la riqueza entre los hogares encuestados. Con los datos de 156 encuestas demográficas y de salud de 66 países, se ha hecho un cálculo de las estimaciones de la riqueza absoluta de los hogares. Se ha validado la metodología comparando la proporción de hogares definidos como pobres según nuestras estimaciones con los informes sobre pobreza publicados por el Banco Mundial. También se ha comparado la precisión de las estimaciones de riqueza absoluta frente a la riqueza relativa para las predicciones de medidas antropométricas.

**Resultados:**

Las estimaciones medias de la riqueza absoluta de 1.403.186 hogares eran 2.056 dólares internacionales per cápita (rango intercuartílico: 723–6.103). La proporción de hogares pobres según las estimaciones de la riqueza absoluta estaba estrechamente relacionada con las estimaciones del Banco Mundial de la población que vive con menos de 2,00 dólares estadounidenses per cápita al día (*R^2^* = 0,84). Las estimaciones de la riqueza absoluta fueron mejores factores predictivos de medidas antropométricas que los índices de riqueza relativa.

**Conclusión:**

Las estimaciones de la riqueza absoluta proporcionan nuevas oportunidades a la investigación comparativa para evaluar los efectos de los recursos económicos en la salud y el capital humano, así como las consecuencias para la salud a largo plazo del cambio económico y la desigualdad.

## ملخص

**الغرض:**

تقدير حجم الثروة المطلق للأسرّ باستخدام بيانات مأخوذة من مسوح ديموغرافية وصحية.

**الطريقة:**

لقد قمنا بتطوير مقياس جديد، وهو تقدير حجم الثروة المطلق، استنادًا إلى ترتيب كل أسرة شملها المسح وفقًا لممتلكاتها المادية والشكل المفترض لتوزيع الثروة بين الأسر التي شملها المسح. باستخدام بيانات ناتجة عن 156 مسحًا من المسوح الديموغرافية والصحية في 66 بلدًا، قمنا بحساب تقديرات حجم الثورة المطلق للأسرّ. وقمنا بتوثيق مصداقية الطريقة من خلال مقارنة نسبة الأسرّ التي تم تحديدها كأسرّ فقيرة من خلال تقديراتنا وباستخدام المؤشرات العددية للفقر المنشورة من البنك الدولي. وقمنا أيضًا بمقارنة تقديرات دقة حجم الثروة المطلق في مقابل حجم الثروة النسبي للتنبؤ بالمقاييس الأنثروبومترية.

**النتائج:**

بلغت تقديرات متوسط حجم الثروة المطلق لـ 1,403,186 أسرة هو 2,056 دولارًا دولية للفرد الواحد (المدى الربيعي: 723–6103). ارتبطت نسبة الأسر الفقيرة استنادًا إلى تقديرات حجم الثروة المطلق ارتباطًا قويًا بتقديرات البنك الدولي للسكان الذين يعيشون بأقل من دولارين أمريكيين للفرد الواحد يوميًا ( *R ^2 ^*= 0.84). وتعد تقديرات حجم الثروة المطلق مؤشرات تنبؤ أفضل للمقاييس الأنثروبومترية بالمقارنة مع مؤشرات الثروة النسبية.

**الاستنتاج:**

توفر تقديرات حجم الثروة المطلق فرصًا جديدة ليقوم البحث المقارن بتقييم تأثيرات الموارد الاقتصادية على الصحة ورأس المال البشري، بالإضافة إلى العواقب الصحية طويلة الأمد للتغيير وعدم المساواة من الناحية الاقتصادية.

## 摘要

**目的:**

旨在通过来自人口与健康调查中的数据估算家庭的绝对财富。

**方法:**

我们依据调查家庭的有形资产和假定的财富分配形态对各个调查家庭进行排名，并据此制定出一项新的指标，即绝对财富估值。通过从 66 个国家中开展的 156 项人口与健康调查中采集数据，我们计算出家庭的绝对财富估值。通过对比界定为贫困的家庭的比例，我们验证了该方法，在使用我们的估值时结合了已公布的世界银行贫困员工人数。我们还针对人体测量的预测对比了绝对和相对财富估值的精确性。

**结果:**

1 403 186 个家庭的绝对财富估算中值为人均 2056 国际元（四分位差：723–6103）。依据绝对财富估值确定的贫困家庭比例与世界银行针对每天人均生活费用低于 2.00 美元 (*R^2^* = 0.84) 的人口发布的估值密切相关。与相对财富指标相比，绝对财富估值是人体测量中比较好的预测指标。

**结论:**

绝对财富估值为对比性研究创造了新机遇，便于我们评估经济资源对健康和人力资本的影响，以及经济变化和不平等问题产生的长期健康后果。

## Резюме

**Цель:**

Оценить абсолютное благосостояние семей с использованием данных демографических опросов и опросов о состоянии здоровья.

**Методы:**

Нами была разработана новая система показателей — оценка абсолютного благосостояния — на основе категории каждой из опрашиваемых семей в соответствии с их материальными ресурсами и предполагаемым характером распределения благосостояния среди опрашиваемых семей. Оценка абсолютного благосостояния семей была рассчитана на основе данных 156 демографических опросов и опросов о состоянии здоровья в 66 странах. Валидация метода проводилась путем сравнения доли семей, охарактеризованных как бедные с использованием нашей оценки, с данными опубликованных отчетов Всемирного банка о численности бедного населения. Кроме того, сравнивалась точность оценок абсолютного и относительного благосостояния для прогнозирования антропометрических показателей.

**Результаты:**

Медианная абсолютная оценка благосостояния в результате исследования 1 403 186 семей составила 2056 международных долларов на человека (межквартильный диапазон: 723–6103). Доля бедных семей, по данным оценки абсолютного благосостояния, сильно коррелировала с оценками Всемирного банка относительно количества населения, живущего менее чем на 2,00 доллара США в день на человека (*R^2^* = 0,84). Абсолютные оценки благосостояния позволяли лучше прогнозировать антропометрические показатели, нежели относительные показатели благосостояния.

**Вывод:**

Абсолютные оценки благосостояния открывают новые возможности для сравнительных исследований, оценивающих воздействие экономических ресурсов на здоровье и человеческий капитал, а также воздействие, которое экономические перемены и неравенство оказывают на здоровье населения в долгосрочной перспективе.

## Introduction

The estimation of the economic status of individuals and their households is central to much work in epidemiology and the social sciences. Wealth is a key determinant of health and social achievement and an indicator of well-being in its own right. For this reason, the development and testing of novel measures of economic status is of interest. There is lively debate over the relative merits of the competing methods used to assess and compare the relative or absolute wealth of individuals and households.^1^^–^^3^

Social scientists have developed several approaches to assess the economic status of households, including consumption expenditures, income, assets and national gross domestic product (GDP) per capita.^1^^,^^2^^,^^4^^–^^6^ The widely used demographic and health surveys have provided detailed data on household assets in over 60 low- and middle-income countries.^3^^,^^7^ Estimates of relative household wealth based on asset data permit researchers to examine the effect of economic status on a wide range of health behaviours and outcomes, such as fertility, growth, malnutrition, disease risk and mortality.^3^^,^^7^^–^^11^ The commonly-used relative wealth indices derived from demographic and health survey data allow households to be ranked according to their relative wealth within a particular country in a particular survey year. However, methods of estimating relative wealth cannot be used to assess the effect of absolute economic resources on health behaviours and outcomes across countries and years. In contrast, estimates of absolute household wealth could be used to make meaningful comparisons across countries and years. They could also be used to compare wealth effects aggregated at multiple social scales – e.g. at country, province, city or household level – and to contribute to current debates about the importance of absolute and relative wealth in determining health outcomes.^5^^,^^12^

Although several methods to estimate absolute household wealth have already been developed or proposed, each has its limitations, including sensitivity to the sample of countries as well as to the country selected as baseline.^13^^,^^14^ Most also rely on arbitrary wealth indicators, cut-offs to anchor comparisons and/or a common set of assets. Such approaches often exclude countries using different assets in surveys, ignore assets that may be important in a specific country setting and assume that an asset in one country provides the same measure of wealth as it does in another country.^13^^–^^15^

In an attempt to address these limitations, we have developed a method for estimating the absolute household wealth per capita – called the absolute wealth estimate – in units that permit meaningful comparisons across countries and years. We used the method to evaluate the prevalence of poverty and indicators of nutritional status and compared these results to common benchmarks.

## Methods

Our method relies on two main inputs from a country in a given year: the rank of each surveyed household according to its material assets and the assumed shape of the distribution of wealth among the surveyed households. Demographic and health surveys provide a ranking of surveyed households, in the form of the wealth factor score.^3^ We used three parameters to define the shape of the wealth distribution in a given country in a given year: (i) the mean wealth per capita; (ii) the Gini coefficient, as a measure of variance; and (iii) the best combination of the Pareto and log–normal distributions that we could identify as a way to estimate skewness.^16^^–^^20^ By using the relative ranking of households by wealth and the shape of the wealth distribution, we could estimate the absolute wealth of each household by identifying the specific rank of the household in the wealth distribution.

### Relative wealth index

The relative wealth index treats household wealth as an underlying, unobserved variable that can be estimated from the presence of household assets and used to assign each surveyed household to one of five wealth quintiles based on rankings on a wealth factor score.^3^ For validation of our method, we used relative wealth indices recorded in demographic and health surveys, belonging to four waves of surveys in 66 countries.

### Mean wealth per capita

We used estimates based on GDP and real per capita consumption data from national accounts (available from the corresponding author).^21^^,^^22^ Our method can use any established measure of country-level wealth per capita.

### Gini coefficient

The distribution of wealth across individuals or households in a given country can be summarized using a Gini coefficient. We obtained the Gini coefficients for wealth from Davies et al.^22^

### Statistical distribution

**Fig. 1:**
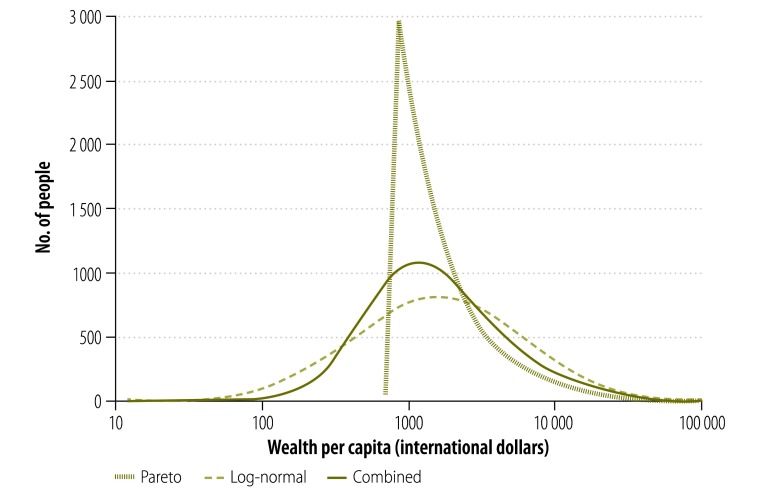
Three possible distributions of wealth per capita, Bangladesh, 2007

Notes: The distributions are based on data collected from 10 997 individuals during a demographic and health survey. The values for wealth are expressed in constant 2011 international dollars, which were based on purchasing power parity exchange rates.

We investigated two statistical distributions commonly used to model wealth in populations: the Pareto and the log–normal. In the modelling of wealth, each of these distributions is defined by the mean wealth per capita – as the central tendency – and the Gini coefficient – as a measure of dispersion. Each distribution has a characteristic shape (Fig. 1). The logarithm of the Pareto distribution is heavily right-skewed, with a sharp cut-off on the left side. The logarithm of the log–normal distribution is a symmetric normal distribution, with no skew. By combining estimates from both of these distributions, we could therefore examine wealth distributions with varying degrees of skew.

### Absolute wealth estimates

Assuming a country-specific wealth distribution, we allocated country-level wealth per capita to each surveyed household based on the household’s wealth factor score ranking. Specifically, the per capita wealth of household *i* was estimated as:


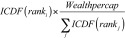
(1)

 where *rank_i_* is the proportional wealth factor score ranking of household *i* and *Wealthpercap* is the country-specific estimate of the wealth per capita in a given year. For the Pareto distribution, *ICDF* is the inverse cumulative distribution function with shape parameter α, calculated as  (1+Gini)/(2 × Gini), and a threshold of [1–(1/α)] × *Wealthpercap*. Otherwise, *ICDF* is the inverse cumulative distribution function for a log–normal distribution based on a normal distribution with a mean of:


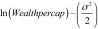
(2)

and a value for σ calculated as:


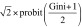
(3)

We called absolute wealth estimates based on Pareto and log–normal distributions Wealth_Pareto_ and Wealth_lognormal_ respectively. Absolute wealth estimates varied – especially among the poorer households – depending on which distribution we used. To examine a wider range of possible distributions with differing degrees of skew, we examined weighted geometric means of the Wealth_Pareto_ and Wealth_lognormal_ values by using the equation:


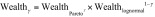
(4)

where γ is the weighting given to a Pareto distribution, relative to the corresponding log–normal distributions, in our calculation of an absolute wealth estimate. The absolute wealth estimate was thus Wealth_lognormal_ when γ was set to zero and Wealth_Pareto_ when γ was set to 1.0. All of the wealth distributions that we considered were therefore defined by three parameters: (i) the mean wealth per capita in a country during the survey year; (ii) the Gini coefficient for wealth; and (iii) γ – i.e. the relative weighting given to the corresponding Pareto and log–normal distributions. All absolute wealth estimates are given in 2011-constant international dollars with purchasing power parity and are therefore comparable over time.

### Validation

We validated our evaluation of absolute wealth in two ways. First, we examined how well the distribution of the estimates from a single country survey approximated a commonly used benchmark for poverty in the same country. The benchmark we used was the World Bank’s so-called poverty headcount – i.e. the World Bank’s estimate of the number of people in a country whose household consumption expenditure, in a given year, fell below 2.00 or 1.25 United States dollars (US$) per day.^23^^,^^24^ Although wealth and consumption expenditure are distinct concepts, we would expect a rough correlation so that, for any given World Bank consumption expenditure used as a threshold for poverty – e.g. US$ 2.00 per capita per day – there should be a comparable threshold value for absolute wealth estimates – e.g. 800 international dollars per capita – that yields a similar estimate of the number of people in poverty. 

Provided that the headcount and demographic and health survey years were separated by no more than three years, we used the World Bank’s poverty headcount for the year closest to the demographic and health survey year. If no World Bank headcount was available for the three years after a survey or three years before a survey, we excluded that survey from our analyses. We used World Bank headcounts based on consumption expenditures from household-level microdata that had been converted to 2005-constant international units. We were able to match 112 headcounts with eligible surveys. We identified the poverty threshold for absolute wealth estimates and the value of γ that produced poverty headcounts that were closest to the corresponding World Bank poverty headcounts. For this purpose, we used threshold consumption expenditures of both US$ 1.25 and US$ 2.00 per capita per day.

In our second method of validation, we compared how accurately different wealth estimates predicted key indicators of nutritional status. We compared the results obtained using our absolute wealth estimates with the corresponding relative wealth estimates. We used demographic and health survey wealth index quintiles as measures of relative wealth. As very few of the surveys we investigated had collected anthropometric data from men, the five key measures were body mass index (BMI) of non-pregnant women aged 20–49 years and the heights and weight-for-height *Z*-scores of boys and girls. 

To assess the relationship between these anthropometric measures and the various wealth estimates, we used a mixed regression model with random effects for country. We classified absolute wealth into 14 categories: less than 200, 200–299, 300–449, 450–679, 680–999, 1000–1499, 1500–2199, 2200–3299, 3300–4899, 4900–7299, 7300–10 999, 11 000–16 499, 16 500–24 999 and at least 25 000 international dollars per capita. The only weight-for-height *Z*-scores that we considered were those of children aged 0–59 months when surveyed and had mothers who were aged 20–49 years when the children were surveyed. The only heights we considered were those of children whose height increase would be expected to be linear with age– i.e. those of children aged 11–59 months.

In the model of BMI, we included a linear and quadratic term for age, a dummy variable for whether a woman was breastfeeding when surveyed,^25^ and interactions between wealth and breastfeeding and wealth and age.^20^ In modelling children’s height, we included an interaction term between wealth and age. In our model of weight-for-height *Z*-scores, we included age in months as a categorical variable and used an age of 24 months for reference. 

To compare the usefulness of our absolute wealth estimates in predicting these key measures of physical growth with that of the wealth quintiles from the demographic and health survey, we ran each model with the estimates alone, with the quintiles alone and with both the estimates and quintiles. The relative fit of each model was assessed using the Akaike information criterion^26^ as well as the proportion of variance accounted for by the absolute wealth estimates and wealth quintiles. We investigated the sensitivity of our method to variation in the Gini estimates and the ranking of households by wealth. We also investigated the sensitivity of the relative wealth analyses to the use of five categories.

## Results

### Descriptives

**Fig. 2:**
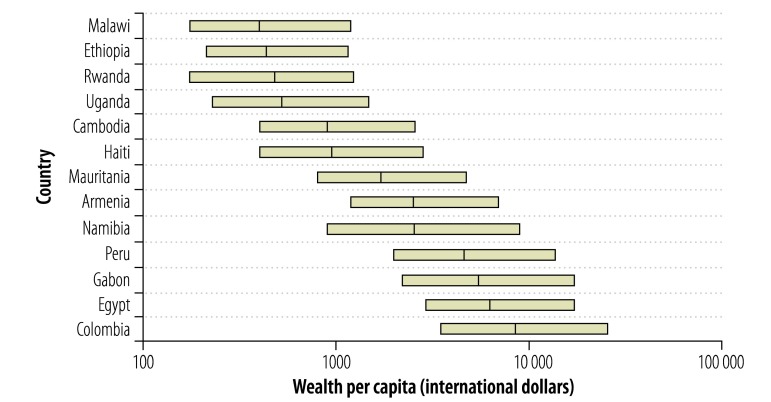
Country ranking by the absolute wealth estimates based on demographic and health surveys conducted in 2000

Notes: The vertical lines and boxes indicate median values and interquartile ranges, respectively. All estimates are expressed in constant 2011 international dollars, which were based on purchasing power parity exchange rates.

**Fig. 3:**
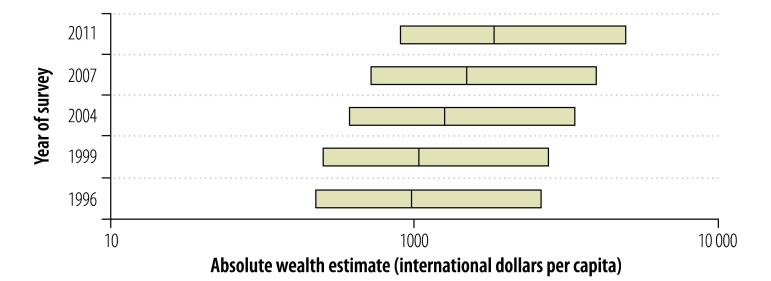
Absolute wealth estimates based on demographic and health surveys, Bangladesh, 1996–2011

Notes: The vertical lines and boxes indicate median values and interquartile ranges, respectively. All estimates are expressed in constant 2011 international dollars, which were based on purchasing power parity exchange rates.

**Fig. 4:**
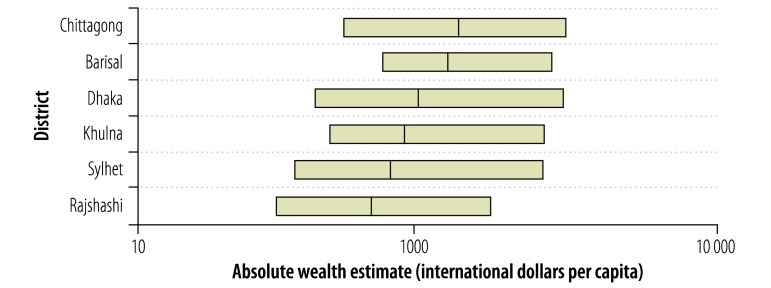
Absolute wealth estimates based on demographic and health surveys in six districts, Bangladesh, 1996

Notes: The vertical lines and boxes indicate median values and interquartile ranges, respectively. All estimates are expressed in constant 2011 international dollars, which were based on purchasing power parity exchange rates.

We analysed wealth data, for 1 989 324 women in 1 403 186 households, collected during 156 surveys in 66 countries. The median absolute wealth estimate was 2056 international dollars per capita (interquartile range: 723–6103). Fig. 2, Fig. 3 and Fig. 4 illustrate the variation seen in absolute wealth estimates across countries in the year 2000 and across time and districts in one country (Bangladesh). Within countries, a uniformly high correlation between the absolute wealth estimates and corresponding relative wealth quintiles was observed (*r* > 0.85).

### Validation

#### Poverty headcounts

**Fig. 5:**
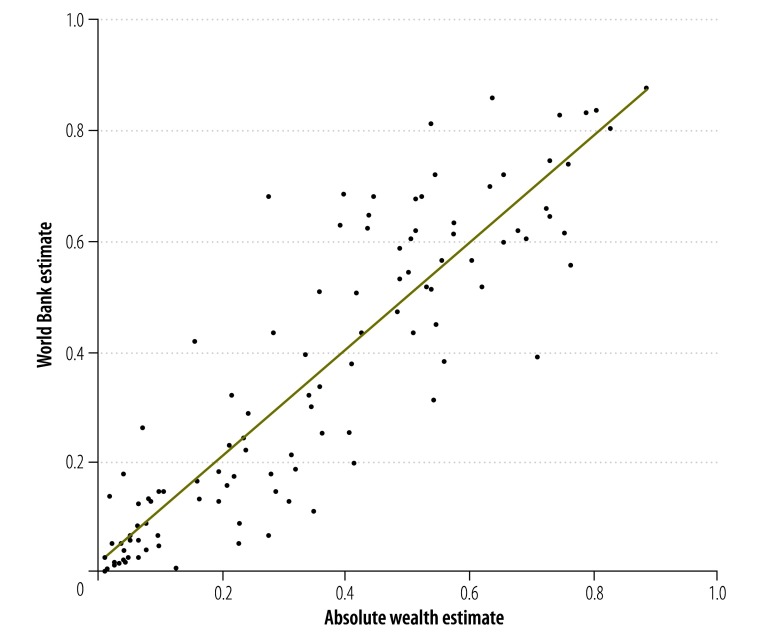
Correlation between two sets of estimates of the proportions of surveyed households in poverty, 66 countries, 1994–2011

Notes: In the World Bank and absolute wealth estimates, households with a daily consumption expenditure below 1.25 constant 2005 international dollars per capita and households with an absolute wealth estimate of less than 1000 constant 2011 international dollars per capita were considered to be in poverty, respectively. Each plotted point is based on data from a demographic and health survey conducted in one of 66 different countries. A trend line has been drawn through the points.

The value of γ that gave the best fit between the poverty headcounts based on the absolute wealth estimates and the World Bank poverty headcounts was 0.32. We therefore used Wealth_0.32_ in subsequent analyses (Fig. 1). The thresholds for Wealth_0.32_ that gave the best approximations of the World Bank poverty headcounts based on thresholds for consumption expenditure of US$ 1.25 and US$ 2.00 per capita per day were about 1000 and 2200 international dollars per capita, respectively. When these thresholds for Wealth_0.32_ were used, there were strong correlations between the poverty headcounts based on the absolute wealth estimates and the corresponding World Bank poverty headcounts: *R^2^* = 0.80 for a threshold of US$ 1.25 per capita per day and *R^2^* = 0.84 for a threshold of US$ 2.00 per capita per day (Fig. 5).

#### Physical growth

All of the anthropometric measures that we investigated showed a statistically significant positive correlation with our absolute wealth estimates. The latter estimates also accounted for substantial proportions of the between-country variation seen in these measures – e.g. 42%, 10–12% and 16–17% of the variation seen in women’s BMI, children’s heights and children’s weight-for-height *Z*-scores, respectively – and smaller proportions – 9%, 4–5% and 1%, respectively – of the corresponding within-country variation. Compared with the relative wealth indexes, the absolute wealth estimates appeared to be much better predictors for the anthropometric measures. The Akaike information criterion values were reduced by 295 and 265 for the heights of boys and girls, respectively, and values were reduced by 2200, 2465 and 34 308 for the weight-for-height *Z*-scores of boys and girls and the BMI of non-pregnant women, respectively. The absolute wealth estimates also accounted for far more of the variance seen in all five of these measures of growth than the corresponding wealth indexes (Table 1). The absolute wealth estimates not only captured at least as much of the within-country relationship between wealth and growth as captured by the wealth indexes but also captured the between-country covariation between wealth and growth (available from corresponding author). The results were robust to assuming a single Gini coefficient, to ranking households instead of individuals by wealth and to using a 14-category relative wealth index instead of the five-category index (available from corresponding author).

**Table 1:** Fit of models predicting anthropometric values from absolute wealth estimates or wealth indexes

Variable	*n*	Proportional reduction of residual error^a^						
		Absolute wealth estimate				Wealth index		
		Between-country	Within-country	Total sample		Between-country	Within-country	Total sample
**Women’s body mass index**	1 109 850	0.42	0.09	0.14		0.00	0.06	0.02
**Height**								
Girls	239 029	0.12	0.04	0.06		0.00	0.03	0.02
Boys	252 416	0.10	0.04	0.06		0.00	0.03	0.02
**Weight-for-height, *Z*-score**								
Girls	306 032	0.16	0.01	0.03		0.00	0.00	0.00
Boys	321 390	0.17	0.01	0.03		0.00	0.00	0.00

^a^ Proportional reduction of residual error in model excluding variable, compared with that in model including the variable. Models used data from 1 403 186 households collected between 1994 and 2011, during 156 demographic and health surveys in 66 countries.

## Discussion

We have developed a method to calculate a novel metric – the absolute wealth estimate – using demographic and health survey data that are regularly collected in many countries. Absolute wealth estimates can be compared across countries and years and with existing measures of relative wealth. This enables researchers to investigate the relationships between absolute economic status and health, fertility, growth and other outcomes of interest. We found that we could derive poverty headcounts from absolute wealth estimates that were consistent with World Bank poverty headcounts. Moreover, the absolute wealth estimates also account for more variation in important measures of human growth and nutrition both within and between countries.

Our method for calculating absolute wealth estimates has several limitations. First, although useful for aggregate analyses, the absolute wealth estimates are likely to have high uncertainty at the level of individual households. Second, given the skewed distribution of wealth and the small number of very wealthy households included in most demographic and health surveys, the absolute wealth estimates for the wealthiest households will be particularly uncertain. We attempted to minimize the impact of such uncertainty by dividing the surveyed households into 14 wealth categories but other researchers may decide to exclude data from households that exceed a relatively high wealth threshold. Third, although we assumed that households in demographic and health surveys had similar social organization, this may not have always been the case since the concept of a household is not clear-cut.^27^^–^^29^

Fourth, the values for country-level wealth per capita that we used relied on estimates of gross domestic products derived from national accounts. National accounts may be inaccurate, especially in poorer countries.^30^ In situations where a large portion of a country’s GDP arises from economic activity that does not contribute to household resources, the wealth available to individual households may be overestimated. In addition, informal economic activity may contribute substantially to household wealth but not be included in estimates of GDP. Finally, the derivation of the relative wealth index assumes that there is a single dominant ladder of wealth. In places with more than one system of wealth – e.g. in areas where cattle ownership may be more important than participation in the cash economy – the wealth score may undervalue household wealth.^31^^,^^32^ We hope that our method for calculating absolute wealth estimates will enable some of these challenges to be resolved and facilitate research on the links between wealth and health. 

A strength of our method to calculate absolute wealth estimates is its dependence on just two main inputs: a relative ranking of households within a survey and the shape of the wealth distribution in the survey population, as defined by three parameters. Unlike other methods for producing comparable estimates of economic status, our method does not depend on the sample of countries or the baseline country selected. In addition, since the relative ranking of households occurs within each country – as a routine part of a demographic and health survey – before estimation of the absolute wealth of the households, our method does not require that all surveys record the same assets or that all assets are weighted the same in terms of their relative importance to wealth in each country. As it enables comparison of wealth estimates without sacrificing information about country-specific assets, it preserves the key strengths of the previous demographic and health survey wealth factor – i.e. the accuracy, efficiency and flexibility of using different asset indicators to measure wealth as a latent variable. Given our method's relative simplicity, it should be possible to extend its use beyond demographic and health surveys, to other nationally representative surveys that also collect data on material assets.

We validated the absolute wealth estimate using anthropometric measures because of the well-established relationship between absolute deprivation and human growth. It appears that, compared with relative wealth, absolute wealth is a much more substantial contributor to at least some of the key measures of human growth and nutritional status. The derivation of absolute wealth estimates may permit researchers to examine how other social and health outcomes – e.g. literacy, cardiovascular risk, infection with human immunodeficiency virus – may be patterned, within countries, provinces and cities, by both absolute and relative wealth.^31^ For example, it may be that literacy is apportioned within countries so that (i) even in the poorest countries, the wealthiest 20% of individuals have high levels of literacy, and (ii) even in the wealthiest countries, the poorest 20% of individuals have low levels of literacy. If this is the case, we would expect relative wealth within countries to be an important, independent predictor of literacy. Absolute wealth estimates provide new avenues for comparing the effects of absolute and within-country relative wealth and investigating their possible interaction.
